# Propofol increases morbidity and mortality in a rat model of sepsis

**DOI:** 10.1186/s13054-015-0751-x

**Published:** 2015-02-19

**Authors:** Martin Schläpfer, Tobias Piegeler, Randal O Dull, David E Schwartz, Mao Mao, Marcelo G Bonini, Birgit Roth Z’Graggen, Beatrice Beck-Schimmer, Richard D Minshall

**Affiliations:** Department of Anesthesiology, University of Illinois at Chicago, 835 S Wolcott Ave (m/c 868), Chicago, IL 60612 USA; Institute of Anesthesiology, University Hospital Zurich, Rämistrasse 100, CH-8091 Zurich, Switzerland; Institute of Physiology, Zurich Center for Integrative Human Physiology, University Zurich, Zurich, Switzerland; Department of Pharmacology, University of Illinois at Chicago, Chicago, IL USA; Department of Bioengineering, University of Illinois at Chicago, Chicago, IL USA; Department of Medicine, University of Illinois at Chicago, Chicago, IL USA; Department of Pathology, University of Illinois at Chicago, Chicago, IL USA

## Abstract

**Introduction:**

Severe sepsis is associated with approximately 50% mortality and accounts for tremendous healthcare costs. Most patients require ventilatory support and propofol is commonly used to sedate mechanically ventilated patients. Volatile anesthetics have been shown to attenuate inflammation in a variety of different settings. We therefore hypothesized that volatile anesthetic agents may offer beneficial immunomodulatory effects during the course of long-term intra-abdominal sepsis in rats under continuous sedation and ventilation for up to 24 hours.

**Methods:**

Sham operation or cecal ligation and puncture (CLP) was performed in adult male Wistar rats followed by mechanical ventilation. Animals were sedated for 24 hours with propofol (7 to 20 mg/kg/h), sevoflurane, desflurane or isoflurane (0.7 minimal alveolar concentration each).

**Results:**

Septic animals sedated with propofol showed a mean survival time of 12 hours, whereas >56% of all animals in the volatile groups survived 24 hours (*P* <0.001). After 18 hours, base excess in propofol + CLP animals (−20.6 ± 2.0) was lower than in the volatile groups (isoflurane + CLP: -11.7 ± 4.2, sevoflurane + CLP: -11.8 ± 3.5, desflurane + CLP -14.2 ± 3.7; all *P* <0.03). Plasma endotoxin levels reached 2-fold higher levels in propofol + CLP compared to isoflurane + CLP animals at 12 hours (*P* <0.001). Also blood levels of inflammatory mediators (tumor necrosis factor-α, interleukin-1β, interleukin-10, CXCL-2, interferon-γ and high mobility group protein-1) were accentuated in propofol + CLP rats compared to the isoflurane + CLP group at the same time point (*P* <0.04).

**Conclusions:**

This is the first study to assess prolonged effects of sepsis and long-term application of volatile sedatives compared to propofol on survival, cardiovascular, inflammatory and end organ parameters. Results indicate that volatile anesthetics dramatically improved survival and attenuate systemic inflammation as compared to propofol. The main mechanism responsible for adverse propofol effects could be an enhanced plasma endotoxin concentration, leading to profound hypotension, which was unresponsive to fluid resuscitation.

**Electronic supplementary material:**

The online version of this article (doi:10.1186/s13054-015-0751-x) contains supplementary material, which is available to authorized users.

## Introduction

Mortality due to severe sepsis has been estimated to be between 28% and 50% [1-6]. Additionally, sepsis has a tremendous impact on healthcare with direct costs of $24.3 billion in 2007 in the United States alone [7]. Despite continuous efforts, therapeutic approaches for sepsis have had limited success. Recently developed drugs to fight sepsis, such as toll-like receptor 4 antagonists [8] and tumor necrosis factor-α (TNF-α) antibodies [9], have also failed to decrease mortality or provide clinical benefit in large phase III studies, and thus there remains a need to develop better treatment strategies.

Eighty-five percent of all septic patients require invasive or non-invasive ventilatory support [10]. Intubation and mechanical ventilation are needed in about 55% to 70% of septic patients admitted to an intensive care unit (ICU) [11], which makes sedation inevitable [12]. Based on this fact, it is crucial to determine the effect and impact of sedatives on the course of sepsis. Traditionally, the use of benzodiazepines combined with opioids has been the standard sedation procedure in the ICU. However, wake-up time can be considerably delayed after a prolonged application of benzodiazepines and therefore, propofol has become a widely-used alternative worldwide [13].

Beneficial immunomodulatory effects of the volatile anesthetic agents sevoflurane [14-20], isoflurane [15,19,21,22] and desflurane [15,20,23] have been demonstrated in various inflammation models. Sedation with volatile anesthetics in the ICU is regarded as an off-label use in many countries [24], although they are often not utilized on a regular basis due to technical limitations [25].

The current study is to our knowledge the first investigation of the combination of mechanical ventilation and continuous sedation over a period of 24 hours in a rodent model of severe sepsis. Most sepsis studies with rodents that included early mechanical ventilation were conducted over a three to six hour period [26-28] that likely did not model the full blown inflammatory response observed at later time points. We hypothesized that volatile anesthetics might reduce morbidity and mortality by attenuating the systemic inflammatory syndrome provoked by cecal ligation and puncture (CLP). This model was chosen because of the clinically relevant condition induced by the polymicrobial infection as compared to ‘sterile’ infection induced by lipopolysaccharide [29]. To mimic ICU conditions, animals were mechanically ventilated continuously with constant sedation induced by either a volatile anesthetic agent (isoflurane, sevoflurane or desflurane) or intravenously applied propofol.

## Methods

### Animals

After approval by the University of Illinois at Chicago Institutional Animal Care and Use Committee (IACUC), pathogen free male Wistar rats (351 ± 47 g) obtained from Charles Rivers (Wilmington, MA, USA) were housed in standard cages with free access to food and water until the time of the experiment.

### Sepsis model, monitoring and ventilation

Anesthesia was induced with ketamine/xylazine applied intraperitoneally (100/10 mg/kg, Ketamine HCl, Hospira Inc., Lake Forest, IL, USA; Xylazine, AnaSed, Akorn Inc., Decatur, IL, USA). Intravenous access for fluid and drug administration was obtained via cannulation of the tail vein with a sterile G24 catheter (BD, Sandy, UT, USA). After shaving and disinfecting the animal’s neck and abdomen, the right carotid artery was dissected and a polyethylene catheter (PE-50) was inserted for blood pressure measurement and repetitive blood sampling, followed by the experimental procedure for the induction of sepsis. The abdominal cavity was exposed by midline incision in all animals. In CLP animals, the cecum was identified and isolated, and 1/3 of the cecum was ligated and punctured once through and through using a 20G needle, which is known to lead to a mid-grade sepsis [29]. In sham animals, no further manipulation was performed before closure of the abdominal wall with sutures.

After the surgical procedure in the abdominal cavity, the trachea was exposed and a polyethylene tube (inner diameter 2.7 mm) was inserted for mechanical ventilation. Animals were ventilated in parallel using pressure-controlled mode (PEEP 4 cmH_2_O, peak inspiratory pressure 16 cmH_2_O, respiratory rate 42 to 47/minute, FiO_2_ 0.35% to 0.45%, using Topo Small Animal Ventilator (Kent Scientific, Torrington, CT, USA)). End-tidal CO_2_ was kept in the normal range (34 to 37 mmHg), adapting respiratory rate accordingly. All surgical sites were closed with two-layers of silk sutures (subcutaneous: running suture, skin: interrupted suture). Sterile saline (0.9%; 0.5 ml/100 g/hour) was administered intravenously for fluid replacement [30]. Arterial blood gases were taken at 0, 6, 12, 18, and 24 hours. Blood levels of liver and kidney markers of organ damage were assessed at 0 and 24 hours (or at time of euthanasia). Body temperature was maintained at 37°C using a warming mat.

### Sedation design

The animals were assigned to one of the following groups: 1) isoflurane-sham (n = 10); sham operation, 24 hour sedation with isoflurane (0.7 to 0.9 vol%, isoflurane, USP, Primal Healthcare, Andhara Pradesh, India, St. Louis, MO); 2) propofol-sham (n = 9); sham operation, 24 hour sedation with propofol (7 to 20 mg/kg/hour, Propoven 1%, Fresenius Kabi, Waltham, MA, USA; median dose: 10.2 mg/kg/hour); 3) isoflurane + CLP (n = 12); CLP, 24 hour sedation with isoflurane (0.7 to 0.9 vol%); 4) sevoflurane + CLP (n = 12); CLP, 24 hour sedation with sevoflurane (1.3 to 1.8 vol% sevoflurane, Baxter, Deerfield, IL, USA); 5) desflurane + CLP (n = 11); CLP, 24 hour sedation with desflurane (4.2 to 5.6 vol%, Suprane, Baxter, Deerfield, IL, USA); and 6) propofol + CLP (n = 12); CLP, 24 hour sedation with propofol (7 to 20 mg/kg/hour; median dose: 7.8 mg/kg/hour).

The following supplementary groups were also studied: 1) isoflurane + intralipid + CLP (n = 9), CLP, 24 hour sedation with isoflurane (0.7 to 0.9 vol%) plus continuous application of intralipid (90 to 110 mg/kg/hour, intralipid 20%, Fresenius Kabi, Uppsala, Sweden); and 2) isoflurane + CLP (12 hour) (n = 9); CLP, 12 hour sedation with isoflurane (0.7 to 0.9 vol%).

Sedation was achieved by delivering 0.6 to 0.8 minimal alveolar concentration (MAC) of the volatile agent (measured by a multi-gas analyzer, Capnomac Ultima, Datex Ohmeda, Helsinki, Finland) or by continuous infusion (with PHD 2000, Harvard Apparatus, Holliston, MA, USA) of 7 to 20 mg/kg/hour propofol. To avoid contamination, a new bottle of propofol was opened for each experiment and syringes were exchanged every eight hours. The level of sedation was titrated to the point where there was restoration of the toe pinch reflex while being able to tolerate mechanical ventilation without distress. Buprenorphine (0.05 mg/kg, Buprenex, Reckitt, Hull, England) was administered subcutaneously at the beginning of the experiment and 12 hours after surgery to provide proper analgesia to the animals. Additional doses of buprenorphine were given to animals showing any sign of not tolerating mechanical ventilation.

At the end of the experiment, the level of anesthesia was deepened with ketamine (50 mg/kg i.v.) and buprenorphine (2.5 mg/kg i.v.), and animals were subsequently euthanized by exsanguination. Plasma was preserved and vital organs were explanted and snap-frozen in liquid nitrogen and stored at −80°C until further analysis.

### Mortality

In the event that mean arterial blood pressure dropped below 60 mmHg for more than 15 minutes, the level of sedation was checked and adapted and additional fluid boluses were administered (up to a cumulative dose of 1 ml/100 g/hour). If blood pressure dropped below 50 mmHg for more than 30 minutes and could not be corrected despite additional fluid resuscitation (humane end-point criteria), the animals were sacrificed for ethical reasons according to IACUC policy.

### Blood gas analysis and serum liver and kidney marker profiles

For assessment of base-excess and oxygenation, 200 μl of whole blood was withdrawn at five different time points (0, 6, 12, 12, 24 hours) into heparinized tubes and analyzed using a GEMPremier 3000 blood gas analyzer (Model 5700, Instrumentation Laboratroy, Lexington, MA, USA). Oxygenation index was calculated as follows: PaO_2_ [mmHg]/FiO_2_.

Determination of liver and kidney markers (aspartate aminotransferase (AST), alanine aminotransferase (ALT), alkaline phosphatase (AP), bilirubin, creatinine, blood urea nitrogen (BUN)) in serum was performed using an Olympus AU400 chemistry analyzer (Olympus, Tokyo, Japan) in the Biological Resources Laboratory core facility at the University of Illinois at Chicago.

### Serum lactate measurement

L-Lactate levels were determined in serum using the L-Lactate Assay Kit (Eton Biosciences, San Diego, CA,USA) according to the manufacturer’s instructions.

### Serum endotoxin measurement

Serum endotoxin levels were determined with the help of the Limulus Amebocyte Lysate (LAL) QCL-1000TM Kit (Lonza, Walkersville, MD, USA). Cross reactivity with propofol and intralipid (intralipid 20%, Fresenius Kabi) was excluded.

### Serum cytokines

TNF-α, interleukin-1alpha (IL-1α), interleukin-1beta (IL-1β), interleukin-6 (IL-6), interleukin-10 (IL-10), C-X-C motif ligand two (CXCL-2) and interferon gamma (IFN-γ) were measured in rat serum with a Luminex performance assay (RnD Systems, Minneapolis, MN, USA) according to the manufacturer’s instructions. Concentration of high mobility group 1 protein (HMGB-1) was assessed by ELISA (IBL international, Hamburg, Germany).

### Serum creatine kinase measurement

We determined creatine kinase (CK) using Fuji Dri-Chem 4000i-Analyzer (FujiFilm, Tokyo, Japan) and Fuji Dri-Chem Slides (CPK-P III, FujiFilm) according to the protocol provided by the company.

### Myeloperoxidase activity in lung, liver, kidney and spleen

Myeloperoxidase (MPO) activity was determined as described previously with minor modifications [31]. The MPOassay buffer contained 0.34 mM H_2_O_2_ and 14.4 mM Guaiacol (both from Sigma-Aldrich, St. Louis, MO) dissolved in phosphate buffered saline, pH 7.4 (PBS, Kantonsapotheke Zürich, Zurich, Switzerland). In the MPO-stop-solution, 2.7 μg catalase (Sigma-Aldrich) was dissolved in 50 ml PBS. Lung, kidney, liver and spleen proteins were extracted as described for heart protein extracts. Five μl of protein extract was added to a 96-well clear microtiter plate (Nunc, Thermo Fisher, Waltham, MA, USA). Three hundred μl MPO reagent was added to each well and stopped with MPO-stop-solution after two minutes of incubation using a multichannel pipette. Absorption of transmitted light was determined at 470 nm (Infinite M200 Pro, Tecan, Männedorf, Switzerland). MPO-activity (A) was then calculated using an extinction coefficient (ε) of 26.6*mM^−1^*cm^−1^ and the formula: A = (dE*V)/(ε*d*v), with A = activity, V = total volume in assay, v = volume of protein extract, d = distance, ε = extinction coefficient, dE = extinction difference = extinction_final_ – extinction_beginning_.

Enzyme activity was corrected for the amount of protein in the extract. Results are presented as U/g tissue protein. One unit is defined as the amount of enzyme consuming 1 μmol H_2_O_2_ per minute.

### Serum nitrate/nitrite (NOx) measurement

One hundred μl rat sera was mixed with 100 μl 10% trichloracetic acid (TCA, Sigma Aldrich) and allowed to sit on ice for 15 minutes in the dark before being centrifuged for 5 minutes at 4°C and 16,100 g. Concentration of serum NO_x_ was determined using a nitric oxide analyzer (NOA^TM280^, Sievers Instruments, Inc., Boulder, CO, USA) as described previously [32,33] .

### Cardiac adenosine triphosphate measurement

Frozen heart tissue was homogenized in radioimmunoprecipitation (RIPA) buffer (Boston BioProducts, Ashland, MA, USA) supplemented with proteinase-inhibitor cocktail, phenylmethanesulfonyl fluoride (200 mM), sodium fluoride (1 mM) and sodium orthovanadate (1 mM), all from Sigma Aldrich (St. Louis, MO, USA) using a Kinematica Polytron homogenizer (Fisher Scientific, Pittsburgh, PA, USA). After 10 brief sonication pulses (Sonic Dismembrator 100, Fisher Scientific, Hampton, NH,USA), the samples were centrifuged for 15 minutes at 4°C and 16,100 g in a microcentrifuge (Eppendorf 5415R, Eppendorf AG, Hamburg, Germany). The supernatant was collected and total protein concentration was determined by DC Protein Assay (Bio-Rad, Hercules, CA, USA) using immunoglobulin G (IgG) (Bio-Rad) for the calculation of the standard curve. Adenosine triphosphate (ATP) levels were determined with an ATP determination kit (Life Technologies, Carlsbad, CA, USA) according to the manufacturer’s protocol.

### Determination of mitochondrial complexes by Western blot

Heart tissue lysates were diluted in 6x Laemmli sample buffer (Boston Bioproducts) to a final concentration of 1x together with dithiothreitol (DTT, 30 μM, Sigma Aldrich). The samples were heated to 50°C for five minutes and 20 μg of protein was loaded on a 10% sodium dodecylsulfate polyacrylamide gel (SDS-PAGE), and then separated and blotted on a nitrocellulose membrane (Bio-Rad) as previously described [34]. After blocking (5% bovine serum albumin, BSA in tris-buffered saline plus 0.1% TWEEN-20, Sigma; TBST) for one hour at room temperature (RT), membranes were probed over night at 4°C with MitoProfile total OXPHOS rodent antibody-cocktail (detecting mitochondrial complexes I-V; Abcam, Eugene, OR, USA, 1:1000). After three five-minute washes with TBST, the membranes were incubated with a horseradish-peroxidase (HRP) conjugated secondary antibody at RT for one hour. After four washes with TBST (five minutes each), HyBlot CL film (Denville, South Plainfield, NJ, USA) was exposed to membranes following incubation with SuperSignal Pico ECL solution (Fisher Scientific). Glyceraldehyde-3-phosphate dehydrogenase protein level (GAPDH, antibody from Santa Cruz Biotechnology, Santa Cruz, CA, USA) was used as a loading control.

### Statistical analyses

Values are expressed as mean (± SD). Statistical analysis was performed with GraphPad Prism 6.0 (GraphPad, La Jolla, CA, USA) and R 3.0.2 (R Development Core Team, R Foundation for Statistical Computing, Vienna, Austria). First, normal distribution of the data was tested with a Shapiro-Wilk test. Comparisons between groups with normally distributed data were carried out using a two-tailed student’s t-test (two groups) or analysis of variance (ANOVA) with Tukey’s *post hoc* test (≥3 groups) or a Mann–Whitney U-Test (two groups), or by Kruskal-Wallis test with Dunn’s multiple comparison correction (≥3 groups) for not-normally distributed data. A *P* value <0.05 was considered to be statistically significant. Survival was analyzed by a log-rank test, heart rate and blood pressure using a mixed linear model with heart rate and blood pressure as response variables, group and time as fixed effects, and individuals as random effects. Multiple comparisons of means in this linear model were performed with a Tukey *post hoc* test.

Samples of animals that had to be sacrificed for ethical reasons (see above) were collected and assigned to the next following time point.

## Results

### Survival

**Figure 1:**
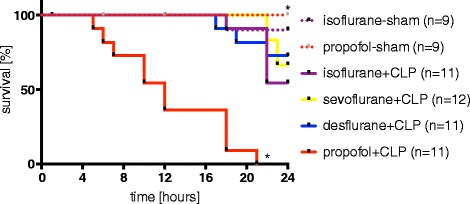
**Survival.** Twenty-four hour survival of rats following cecal ligation and puncture (CLP) or sham operation under continuous sedation with propofol, isoflurane, sevoflurane or desflurane and continuous mechanical ventilation. Survival was compared using the Log-rank test, *P <0.05 versus isoflurane + CLP.

Ninety-four percent (17 out of 18) of animals in the sham groups (isoflurane-sham and propofol-sham) survived the entire 24-hour experimental period. In septic animals (CLP) sedated with any of the three volatile agents, overall survival rate was 65% (22 out of 34). Survival in the isoflurane + CLP (55%, 7 out of 11), sevoflurane + CLP (67%, 8 out of 12) and desflurane + CLP (73%, 8 out of 11) groups was not significantly different from the two sham groups. However, mean survival of septic animals sedated with propofol (CLP-propofol) at 12 hours was significantly reduced compared to all other groups (*P* <0.001, Figure 1). Intralipid application did not affect mortality in isoflurane + CLP animals (Additional file 1).

### Development of metabolic acidosis

**Figure 2:**
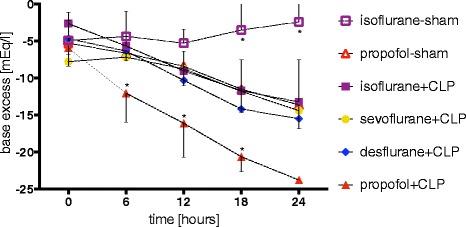
**Acidosis.** Development of acidosis, measured by base excess after 0, 6, 12, 18 and 24 hours in septic and sham-operated rats. Animals were under continuous sedation with propofol, isoflurane, sevoflurane or desflurane and under continuous mechanical ventilation. Values represent mean ± standard deviation. *P <0.05 versus isoflurane + CLP. CLP, cecal ligation and puncture.

Base excess (BE) rather than pH was chosen in order to correct for possible ventilation differences between the animals. In all CLP groups as well as in the propofol-sham group, BE decreased. Development of acidosis in the propofol + CLP group was faster and more pronounced when compared to all other groups (Figure 2). At 6, 12 and 18 hours, BE was significantly lower in the propofol + CLP group in comparison to all septic groups sedated with volatile anesthetics (6 hours: −12.0 ± 3.9 mEq/L, all *P* <0.01, 12 hours: −16.1 ± 4.6 mEq/L, all *P* <0.001 and 18 hours: −20.7 ± 2.0 mEq/L, all *P* <0.01, respectively). One animal surviving close to 24 hours in the propofol + CLP group had a BE of −23.8 mEq/L. Application of intralipid had no influence on the development of acidosis (Additional file 2).

Interestingly, sham-operated animals sedated with propofol developed acidosis as well, whereas animals sedated with isoflurane did not (Figure 2). These BE differences were statistically significant after 18 and 24 hours (−11.5 ± 3.1 versus -3.4 ± 4.1 and −13.6 ± 3.2 versus -2.4 ± 3.9, *P* <0.001 for both comparisons).

Lactate in isoflurane-sham animals was 1.08 ± 0.77 mM after 24 hours of sedation. This value was significantly lower than in the propofol-sham (2.50 ± 0.46 mM) and isoflurane + CLP (2.25 ± 1.24 mM) animals (*P* = 0.001 and 0.004, respectively). Serum lactate also reached higher values in propofol + CLP compared to isoflurane + CLP animals after 12 hours (3.03 ± 0.42 versus 1.33 ± 0.31 mM, *P* <0.001, Additional file 3).

### Oxygenation

The oxygenation index was not significantly altered over the course of the experiment in any of the experimental groups (Additional file 4).

### Heart rate

**Figure 3:**
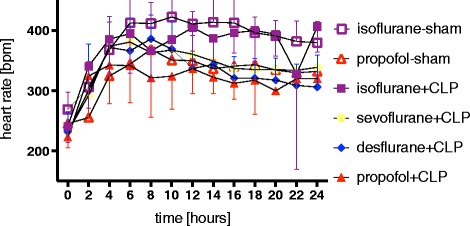
**Heart rate.** Effect of continuous sedation with propofol, isoflurane, sevoflurane or desflurane on heart rate (beats per minute, bpm) in septic (CLP) and sham-operated rats. Values represent mean ± standard deviation. CLP, cecal ligation and puncture.

No difference in heart rate was observed between septic and sham-operated animals. However, heart rate was consistently higher in all isoflurane-sedated animals: isoflurane-sham animals had an average heart rate of 383 ± 57 beats per minute (bpm) versus propofol-sham animals 328 ± 47 bpm (*P* <0.001). The same range of values was observed in septic animals: the isoflurane + CLP group presented with a heart rate of 372 ± 69 bpm compared to the propofol + CLP (313 ± 64 bpm; *P* <0.001), sevoflurane + CLP (339 ± 62 bpm; *P* = 0.01), and desflurane + CLP (330 ± 60 bpm; *P* = 0.01) animals, respectively (Figure 3 and Additional file 5).

### Blood pressure

**Figure 4:**
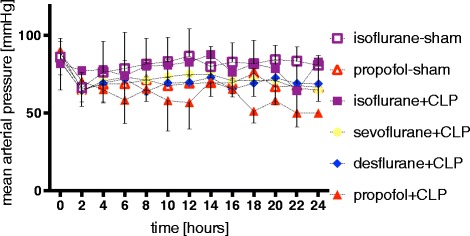
**Blood pressure.** Effect of continuous sedation with propofol, isoflurane, sevoflurane or desflurane on mean arterial blood pressure in septic (CLP) and sham-operated rats. Values represent mean ± standard deviation. CLP, cecal ligation and puncture.

Overall, as shown in Figure 4 and Additional file 6, lower blood pressure values could be found in desflurane + CLP (70 ± 12 mmHg) and propofol + CLP (67 ± 16 mmHg; *P* = 0.03) rats compared to isoflurane + CLP animals (79 ± 18 mmHg; *P* <0.01 for both comparisons).

### Intravenous fluid administration and hematocrit values

Isoflurane-sham and propofol-sham animals received similar amounts of NaCl over the course of the experiment (23.5 ± 2.2 ml versus 23.9 ± 2.4 ml). Fluid administration in septic animals was significantly higher (42.9 ± 14.5 ml, 38.8 ± 15.7 ml and 53.7 ± 20.5 ml for isoflurane + CLP, sevoflurane + CLP and desflurane + CLP, *P* = 0.005, 0.03 and *P* <0.001 versus isoflurane-sham). Propofol + CLP animals had an even higher fluid requirement (60.2 ± 17.0 ml versus 11.8 ± 3.5 ml after 12 hours compared to isoflurane + CLP, *P* <0.001). Hematocrit values at the end of the experiment were similar in all animal groups with values of 24.3 ± 3.9 (isoflurane-sham), 24.5 ± 2.5 (propofol-sham), 24.2 ± 4.4 (isoflurane + CLP at 24 hours), 23.0 ± 2.1 (isoflurane + CLP at 12 hours), 23.2 ± 3.5 (sevoflurane + CLP), 26.7 ± 3.2 (desflurane + CLP) and 23.2 ± 3.5 (propofol + CLP).

### Kidney and liver damage marker profiles

ALT was increased in the sevoflurane + CLP (59.3 ± 24.7 U/l) and the isoflurane + CLP (68.5 ± 38.7 U/l) groups compared to sham-operated isoflurane-treated animals (33.8 ± 7.6 U/l), *P* = 0.03 and *P* = 0.03, respectively; Table 1). No differences in AST were detected. After 12 hours, AP in propofol + CLP treated animals was 264 ± 185 U/l compared to 187 ± 46 U/l in isoflurane + CLP animals (P <0.001, Table 1).

**Table 1:** **Serum liver and kidney marker profile at 24 and 12 hours**

	**AP [U/l]**	**ALT [U/l]**	**AST [U/l]**	**Bilirubin [mg/dl]**	**BUN [mg/dl]**	**Creatinine [mg/dl]**
**24-hour animals (sham and CLP)**						
isoflurane-sham	126(± 20)	33.8(± 7.6)	93(± 33)	0.109(± 0.027)	38.8(± 14.8)	0.33(± 0.13)
propofol-sham	367(± 161)***	35.6(± 6.1)	78(± 17)	0.176(± 0.055)*	59.4(±12.0)	0.72(± 0.32)
Isoflurane +CLP	253(± 117)*	68.5(± 38.7)*	210(± 177)	0.208(± 0.089)**	71.4(± 21.2)*	0.80(± 0.28)*
Sevoflurane + CLP	232(± 53)**	59.3(± 24.7)*	172(± 107)	0.186(± 0.073)*	73.1(± 33.0)**	0.88(± 0.52)**
Desflurane + CLP	262(± 60)***	42.1(± 16.1)	107(± 55)	0.151(± 0.024)	66.7(± 17.0)*	0.86(± 0.38)*
**12-hour CLP animals**						
Isoflurane + CLP	187(± 46)	73.6(± 34.3)	222(± 136)	0.164(± 0.051)	58.6(± 12.7)	0.53(± 0.19)
propofol + CLP	264(± 185)^##^	67.3(± 42.2)	168(± 109)	0.255(± 0.104)^#^	43.3(± 22.4)	0.85(± 0.47)

*P <0.05, **P <0.01, ***P <0.001 versus sham-isoflurane; ^#^ *P* <0.05, ^##^ *P* <0.001 versus isoflurane + CLP 12 hour. ALT, alanine aminotransferase; AP, alkaline phosphatase; AST, aspartate aminotransferase; BUN, blood urea nitrogen; CLP, cecal ligation and puncture.

After 24 hours, bilirubin was increased in propofol-sham animals (0.176 ± 0.055 mg/dl) compared to isoflurane-sham (0.109 ± 0.027 mg/dl; *P* = 0.03, Table 1). Blood urea nitrogen and creatinine were higher in all septic compared to isoflurane-sham animals (all *P* <0.05, Table 1).

### Blood endotoxin concentration

**Figure 5:**
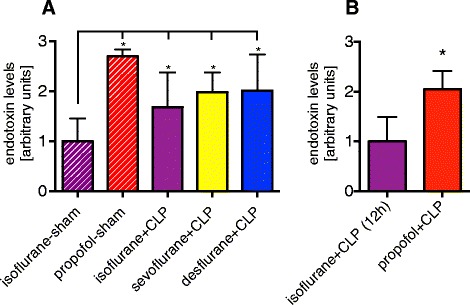
**Blood endotoxin concentration.** Effect of continuous sedation with propofol, isoflurane, sevoflurane or desflurane on blood endotoxin levels in septic (CLP) and sham-operated rats. Animals were under continuous sedation and mechanical ventilation for 24 hours **(A)** or 12 hours **(B)**. Values represent mean ± standard deviation. *P <0.05 versus isoflurane-sham **(A)** or isoflurane + CLP **(B)**, respectively. CLP, cecal ligation and puncture.

In septic animals, a 1.7-, 2.0- and 2.0-fold increase of endotoxin concentration in the blood was observed after 24 hours in isoflurane, sevoflurane and desflurane-treated rats (*P* = 0.05, 0.001 and 0.001 versus isoflurane-sham, respectively; Figure 5A). Propofol-sham animals had 2.7-times higher endotoxin levels in their blood compared to the isoflurane-sham group (*P* <0.001, Figure 5A). Intralipid increased blood endotoxin levels by 42% in isoflurane + intralipid + CLP versus isoflurane + CLP animals (*P* = 0.01, Additional file 7). At 12 hours, endotoxin concentration increased two-fold in septic animals sedated with propofol compared to those sedated for 12 hours with isoflurane (*P* <0.001, Figure 5B).

### Blood cytokines

Blood cytokine levels were higher in septic animals. In rats sedated with propofol, even more cytokines were found. TNF-α was increased in the propofol-sham (89 ± 56 pg/ml), isoflurane + CLP (282 ± 291 pg/ml), and sevoflurane + CLP (89 ± 70 pg/ml) groups compared to isoflurane-sham (23 ± 15 pg/ml; *P* = 0.01, *P* <0.001 and *P* = 0.03, respectively). Increased values for IL-1β, IL-6, IL-10 and CXCL-2 were also observed in all volatile anesthetic sepsis groups compared to isoflurane-sham animals (all *P* <0.03, Table 2), and IFN-γ was increased in isoflurane + CLP compared to isoflurane-sham animals (134.2 ± 131.9 versus 12.4 ± 12.2 pg/ml, *P* = 0.04; Table 2). After 12 hours, septic animals sedated with propofol showed increased levels of TNF-α, IL-1β, IL-10, CXCL-2, IFN-γ and HMGB-1 compared to animals sedated with isoflurane (all *P* <0.05; Table 2).

**Table 2:** **Serum cytokines at 24 and 12 hours**

	**TNF-α [pg/ml]**	**IL-1α [pg/ml]**	**IL-1β [pg/ml]**	**IL-6 [ng/ml]**	**IL-10 [pg/ml]**	**CXCL2 [ng/ml]**	**IFN-γ [pg/ml]**	**HMGB-1 [ng/ml]**
**24 hour animals (sham and CLP)**								
Isoflurane-sham	22.7(± 14.6)	13.9(± 9.2)	143(± 125)	4(± 3)	151(± 95)	0(± 0)	12.4(± 12.2)	0.0(± 0.0)
Propofol-sham	88.7(± 55.9)*	16.7(± 10.3)	368(± 112)	46(± 23)	605(± 265)	2(± 2)	79.3(± 119.4)	0.0(± 0.0)
Isoflurane + CLP	281.9(± 291.3)***	94.9(± 124.7)	510(± 418)*	220(± 179)***	1717(± 1567)***	38(± 42)***	134.2(± 131.9)*	13.5(± 22.3)
Sevoflurane + CLP	89.1(± 70.5)*	30.6(± 29.1)	459(± 219)**	65(± 33)**	774(± 402)**	15(± 13)***	50.5(± 53.3)	4.0(± 7.2)
Desflurane + CLP	61.8(± 31.0)	42.9(± 54.5)	537(± 325)**	69( 32)**	781(± 369)**	24(± 17)***	83.4(± 93.6)	0.0(± 0.0)
**12 hour CLP animals**								
Isoflurane + CLP	141.4(± 78.8)	24.0(± 17.8)	325(± 217)	45(± 16)	267(± 109)	3(± 3)	3.6(± 0.0)	0.0(± 0.1)
Propofol + CLP	549.9(± 440.2)	165.8(± 196.1)^###^	1446(± 954)^###^	126(± 117)	1240(± 1148)^#^	41(± 55)^#^	541.9(± 567.7)^#^	10.1(± 10.7)^#^

*P <0.05, **P <0.01, ***P <0.001 versus sham-isoflurane; ^#^ *P* <0.05, ^###^ *P* <0.001 versus isoflurane + CLP 12 hour. CLP: cecal ligation and puncture; CXCL2: chemokine (c-x-c motif) ligand 2; HMGB-1: high mobility group protein-1; IFN-γ: interferon gamma; IL-1α: interleukin-1alpha; IL-1β: interleukin-1beta; IL-6: interleukin-6; IL-10: interleukin-10; TNF-α: tumor necrosis factor-alpha.

### Creatine kinase values

To exclude propofol infusion syndrome, serum CK levels were determined. Due to high inter-individual differences, no significant difference could be detected in the 24 hour-animals. Values from sham animals seemed to be lower than from CLP groups (Additional file 8: Figure A).

At 12 hours, CK was similar in the CLP groups: 1115 ± 786 U/L in isoflurane + CLP and 996 ± 838 U/L in propofol + CLP animals (Additional file 8: Figure B).

### Tissue neutrophil content in lung, spleen, kidney and liver

Sepsis increased MPO activity in the lung, but the observed difference only reached statistical significance between the isoflurane + CLP and the isoflurane-sham groups (1.7-fold increase, *P* = 0.02). Also, propofol by itself increased MPO activity in the lung: propofol-sham animals revealed a 2.3-fold increase in MPO activity compared to isoflurane-sham (*P* <0.001), and after 12 hours, CLP-propofol animals showed a 1.9-fold increase in MPO activity compared to isoflurane + CLP animals (*P* <0.001, Table 3).

**Table 3:** **MPO activity various tissues**

**MPO activity [U/g]**	**Lung**	**Kidney**	**Spleen**
**24 hour animals (sham and CLP)**			
isoflurane-sham	27.08(± 10.23)	1.20(± 0.39)	4.77(± 2.56)
propofol-sham	63.03(± 13.34)***	1.40(± 0.64)	10.12(± 6.75)*
isoflurane + CLP	44.79(± 18.11)*	2.07(± 0.67)	4.77(± 2.56)
sevoflurane + CLP	41.27(± 8.68)	1.53(± 0.57)	7.35(± 4.25)
desflurane + CLP	38.00(± 9.90)	0.87(± 0.24)	8.61(± 3.23)
**12 hour CLP animals**			
isoflurane + CLP	28.48(± 11.42)	0.78(± 0.26)	5.94(± 3.51)
propofol + CLP	54.20(± 9.75)^###^	0.91(± 0.21)	7.17(± 3.90)

*P <0.05, ***P <0.001 versus sham-isoflurane; ^###^ *P* <0.001 versus isoflurane + CLP 12 hour. CLP: cecal ligation and puncture; MPO: myeloperoxidase.

In the spleen, MPO activity was 2.1-times higher in the propofol-sham versus isoflurane-sham animals at 12 hours (*P* = 0.04, Table 3). No differences were observed in the kidney. In the liver, MPO activity was below the limit of detection.

### Blood NO_x_ values

Similar levels of NO_x_ were found in the blood of all animals. The values for the different groups after 24 hours were 73.88 (±15.85), 25.16 (±9.72), 68.36 (±51.56), 17.91 (±6.15) and 251.80 (±141.50) for isoflurane-sham, propofol-sham, isoflurane + CLP, sevoflurane + CLP and desflurane + CLP, respectively (Additional file 9).

### Cardiac ATP content

ATP levels in cardiac tissue lysates were not significantly different among the various treatment groups.

### Cardiac mitochondria complexes

In the 24-hour groups, no differences in expression of mitochondrial complexes were detected. Similar results were found after 12 hours. However, while no differences in mitochondrial complexes I, II, III and V were observed, complex IV (cytochrome c oxidase) was increased by 3.3 (±1.6) fold in propofol + CLP compared to isoflurane + CLP animals after 12 hours of sedation (*P* = 0.03, Additional file 10).

## Discussion

### Long-term sepsis model

With this work, we describe a newly developed experimental model of rodent sepsis lasting up to 24 hours with sedation and mechanical ventilation. The current study provides compelling evidence to suggest that: 1) sedation of septic rats with volatile anesthetics (isoflurane, sevoflurane or desflurane) tremendously reduces mortality compared to rats sedated with propofol, and 2) in the setting of prolonged intra-abdominal sepsis, propofol may actually hasten physiological deterioration. Sedation with volatile anesthetics leads to a slower development of acidosis, reduced expression of pro-inflammatory cytokines, and reduced levels of plasma endotoxin compared to sedation with propofol.

Our model employed continuous sedation, along with mechanical ventilation, arterial blood pressure and heart rate monitoring, as well as fluid resuscitation, and, thus, it very closely resembled the clinical situation in an ICU. The long observation time not only allowed for determination of surrogate markers, but also the evaluation of a strong outcome parameter such as survival, which is the most important aspect within the clinical picture of severe polymicrobial sepsis.

### Sedation and base excess

In interpreting the mortality data, the question of cause of death arises. One explanation could be the more accentuated impairment of the acid base balance in septic animals. Comparing the propofol + CLP group with animals sedated with volatile anesthetics, it is evident that BE is significantly worse in the presence of propofol. The hemodynamic situation as another important factor likely influencing survival cannot be interpreted, as low blood pressure values were counteracted with a pre-defined fluid resuscitation protocol. Therefore, one could assume that metabolic acidosis as a consequence of sepsis in the propofol + CLP group further accentuated the persistent propofol-induced vasoplegia leading to severe hypotension with subsequent organ hypoperfusion and, finally, malfunction of critical organs [35]. A relative ‘intoxication’ with propofol in comparison to the volatile anesthetics can be excluded as depth of sedation was carefully observed and adjusted using indirect measures. Therefore, the same depth of anesthesia can be assumed in all animal groups. Also, occurrence of propofol infusion syndrome seems rather unrealistic as serum CK values were comparable in both sedation groups.

Interestingly, propofol reduced BE independent of the presence of sepsis. A similar phenomenon was observed in pediatric patients undergoing heart catheterization where propofol led to a decrease in BE and pH without a rise in blood lactate levels [36]. Ypsilantis *et al*. reported more pronounced acidosis and 100% mortality in healthy rabbits upon an aimed 48 hour exposure to propofol compared to sevoflurane [37].

### Intralipid and base excess

As a next step, we carefully tested the hypothesis that the lipid emulsion *per se* could be the causative agent in the intravenous propofol solution. However, data clearly showed that administration of intralipid alone did not correlate with metabolic acidosis. Propofol infusion syndrome could be discussed as well, although reported serum values of lactate for this disease in humans are much higher than the values we observed in our study [38]. Therefore, we can conclude that propofol itself, by inducing metabolic acidosis as a first hit, may set the stage for a second injury such as sepsis that can result in extreme deterioration of the acid–base balance and finally death of the animals.

As indicators for circulation, we monitored heart rate and blood pressure continuously. As rats are prone to right heart failure, continuous PEEP was applied and recruitment maneuvers were conducted every thirty minutes [39]. Thus, in our experiment, animals may have died from insufficient circulation due to hypotension as a result of heart failure, vasoplegia or hypovolemia. ATP levels were similar in all groups and a fluid replacement protocol was followed; therefore, we would predict that the cardiac energy supply was not a limiting factor for survival. Acidosis, however, may affect cardiac function [40,41] and tone of the vascular system [42] resulting in low output failure and relative hypovolemia.

### Volatile anesthetics and organ dysfunction in sepsis

With regard to mortality, there were no differences among the volatile anesthetics tested. Considering surrogate markers such as inflammatory mediators, however, one could argue that sevoflurane may be superior to desflurane and isoflurane, although larger studies aimed at testing this hypothesis would be needed.

According to the liver and kidney serum markers of organ damage obtained from the animals in our study, mortality was not primarily caused by acute liver or kidney failure. However, it is interesting to note that that AST and ALT levels were higher in sevoflurane and isoflurane treated animals compared to desflurane treated animals. Inorganic fluoride ion concentrations are known to increase during isoflurane [43] and sevoflurane [44] anesthesia compared to desflurane anesthesia, which may have had an impact on liver function as well as on AST and ALT release [45].

Previous studies have reported a reduction of cytokines in septic animals under the influence of isoflurane [20,22,46,47], sevoflurane [19,20] and desflurane [20,23] which we also observed. Interestingly, there are several studies showing possible anti-inflammatory effects of propofol [48-50]. However, most of these studies did not directly compare propofol exposure to treatment with a volatile anesthetic agent [48,50]. It is also not clear in these studies which propofol formulation was used [48,51] or whether it contained ethylenediaminetetraacetic acid (EDTA) [49,50] which is known to account for many of the observed anti-inflammatory effects of propofol due to its ability to destabilize the bacterial wall [52]. As our goal was to investigate the potential immunomodulatory effect of sedatives and not of additives, we utilized a propofol formulation without EDTA in our study. Interestingly, HMGB-1, a cytokine with a negative predictive value on survival in sepsis [53], was indeed significantly increased in propofol + CLP rats, that is, in the animals that actually succumbed earlier as compared to isoflurane + CLP animals.

### Plasma endotoxin and innate immunity

The elevated level of endotoxin in propofol-sedated rats compared to the volatile anesthetic groups suggests that propofol may increase host susceptibility to infection, suppress bacterial clearance, or increase the release of lipopolysaccharide (LPS) into the bloodstream after increased bacterial translocation or growth. Another consideration is that bacterial growth may be enhanced by lipid formulations, a phenomenon which is known to be prevented in the presence of EDTA in the propofol formulation [54]. To avoid contamination, strict aseptic technique was applied during our experiments. For each experiment, a new bottle of propofol was used and propofol syringes were replaced every eight hours. Given the overall higher levels of serum cytokines in propofol-treated animals, our results suggest that the reaction of the animals’ immune system remained intact and that more bacteria may have led to more endotoxin and a more pronounced cytokine storm. [55] However, this does not exclude a propofol effect on innate immunity. Activation of inducible NO-synthase (iNOS) may lead to an increase in superoxide production [56] which is considered to be an important defense mechanism against bacteremia, whereas inhibition of iNOS may exacerbate an infection [57]. It has been suggested that propofol exposure may inhibit early superoxide production in neutrophils [58]. If this had been the reason for the increase in mortality in our setting, we would have expected lower values of serum nitric oxide (NO). However, serum concentrations of NO_x_ did not differ significantly among the groups, and, thus, NO_x_ is likely not responsible for the defect in immune defense or for promoting bacterial growth in animals sedated with propofol.

The effect of propofol on gut barrier function in septic or in healthy subjects is not known. Long-term application of intravenous lipid solutions – the carrier substrate for propofol – however, reduces intestinal cell proliferation, increases intestinal epithelial apoptosis and reduces gut barrier function [59]. This might explain the increased endotoxin levels in our animals exposed to intralipid. Integrity of the intestinal barrier may have been further reduced by acidosis [60] due to propofol application. We, therefore, hypothesize that the gut barrier might be markedly impaired in the presence of propofol. A recent study suggests that propofol might have a protective effect on the gut barrier in rats following burn injury [61]. However, in this particular study, direct comparison of propofol and volatile anesthetics was not performed and, further, it is not clear which propofol formulation was used.

Our study does not clearly answer the question whether we are able to show improved survival by volatile anesthetics due to positive immunomodulation or if we are demonstrating detrimental effects of propofol on microcirculation. It is described that propofol affects microcirculation, for example, in the gastric mucosa [62]. It is further known that differences in microcirculation account for organ failure and eventually also death in septic patients [63]. Maybe a second intravenous agent, such as midazolam, could have addressed this issue. Despite these open questions, volatile anesthetics seem to be the better option in our model.

We are aware that performing sedation with volatile anesthetics is an off-label use in many countries and is not considered a standard regimen [24]. However, these drugs may provide benefits such as no organ-dependent degradation, minimal metabolism and short titration and awakening times [64].

A definite strength of our study design was the direct comparison of the effect of clinically relevant sedatives with volatile anesthetics on the course of sepsis. This is the first study to compare long-term application of sedatives during simultaneous mechanical ventilation, and thus, is more likely to reflect the situation of a septic patient in the ICU than studies comparing short-term application of sedatives before or after onset of an injury. To limit the number of variables in the current study, we omitted the use of antibiotics and vasopressors, which at the same time could be a limitation of our study. A further limitation of this study might be the fact that rats need higher doses of propofol to achieve light sedation compared to humans [65], probably due to higher propofol clearance rates [66], whereas the MAC values of volatile anesthetics are similar [67].

## Conclusions

In summary, the results of our study may be of significant clinical relevance as they further support volatile anesthetics as the sedative agent of choice for septic patients. Greater implementation of the use of volatile agents in the ICU setting may significantly affect the course of inflammation, the microbial load, and potentially, the survival of septic patients who currently exhibit extremely high mortality rates. Clinical trials are needed to confirm these results.

## Key messages

- This is the first model of prolonged mechanical ventilation in severe sepsis (up to 24 hours) comparing beneficial and detrimental effects of various sedative strategies
- The commonly used clinical sedative propofol is associated with increased blood endotoxin levels, a stronger inflammatory reaction, a more pronounced acidosis and, importantly, with poor survival.

## Additional files

Additional file 1:
**Survival of isoflurane + intralipid + CLP animals.**
Additional file 2:
**Base excess of isoflurane + intralipid + CLP animals.**
Additional file 3:
**Serum lactate in sham-operated and CLP-animals after 24 and 12 hours.**
Additional file 4:
**Blood oxygenation index (Horowitz index, (mmHg), mean ± standard deviation).**
Additional file 5:
**Individual time trajectories of heart-rate in sham-operated and CLP-animals.**
Additional file 6:
**Individual time trajectories of mean arterial pressure in sham-operated and CLP-animals.**
Additional file 7:
**Influence of intralipid on serum endotoxin levels.**
Additional file 8:
**Influence of sedatives on creatine kinase in sepsis.**
Additional file 9:
**Serum NOx levels in sham-operated and CLP-animals.**
Additional file 10:
**Amount of mitochondrial complex IV in septic animals after 12 hours.**

## Abbreviations

ALT: alanine aminotransferase
AP: alkaline phosphatase
AST: aspartate aminotransferase
ATP: adenosine triphosphate
BE: base excess
bpm: beats per minute
BSA: bovine serum albumin
BUN: blood urea nitrogen
CK: creatine kinase
CLP: cecal ligation and puncture
CXCL-2: C-X-C motif ligand two
DTT: dithiothreitol
ELISA: enzyme-linked immunosorbent assay
HMGB-1: high mobility group protein-1
HRP: horseradish peroxidase
IACUC: Institutional Animal Care and Use Committee
IFN-γ: interferon gamma
IL-10: interleukin-10
IL-1β: interleukin-1beta
IL-1α: interleukin-1alpha
IL-6: interleukin six
MAC: minimum alveolar concentration
MPO: myeloperoxidase
NO_x_: nitrogen oxide
PAGE: polyacrylamide gel
PBS: phosphate-buffered saline
RIPA: radioimmunoprecipitation assay
RT: room temperature
SD: standard deviation
SDS: sodium dodecyl sulfate
TBST: Tris buffered saline with tween
TNF-α: tumor necrosis factor-alpha
