# Supplementary material for: Propofol increases morbidity and mortality in a rat model of sepsis
Source: Crit Care. 2015 Feb 19;19(1):45. doi: 10.1186/s13054-015-0751-x (PMC4344774; doi:10.1186/s13054-015-0751-x)
Supplement: Additional file 2: — Base excess of isoflurane + intralipid + CLP animals. [file 13054_2015_751_MOESM2_ESM.pdf]

Additional file 2

Base excess of isoflurane+intralipid+CLP animals

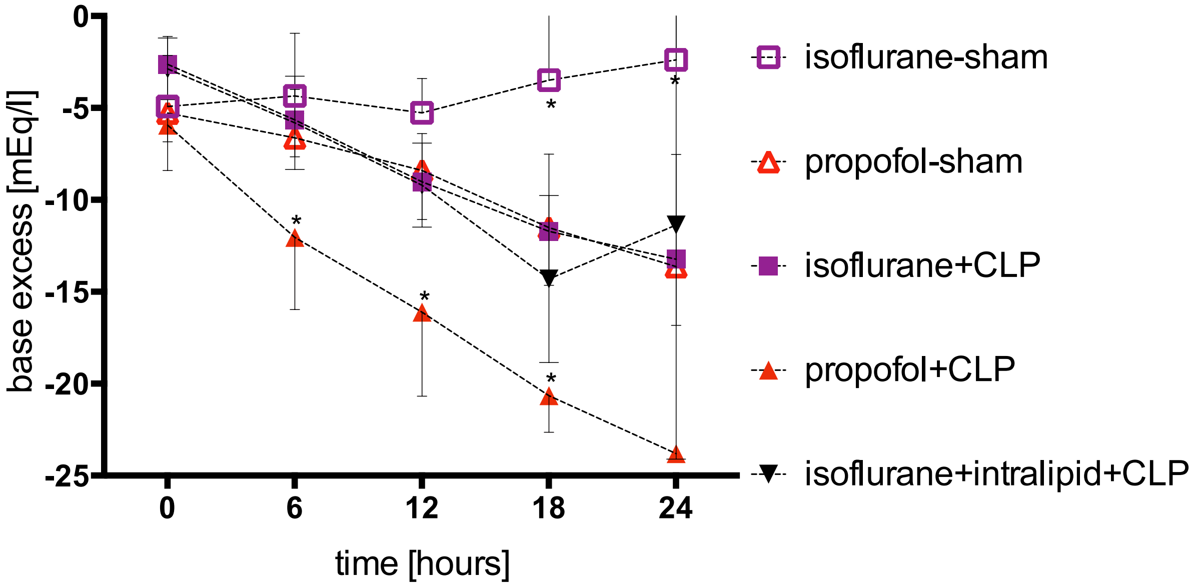

Exclusion of detrimental effects of intralipid on development of acidosis, measured by base excess, in septic (CLP) and sham-operated rats under continuous sedation with propofol or isoflurane and continuous mechanical ventilation. Values represent mean ± standard deviation. *$p<0.05$ vs. isoflurane+CLP.
