# Supplementary material for: Propofol increases morbidity and mortality in a rat model of sepsis
Source: Crit Care. 2015 Feb 19;19(1):45. doi: 10.1186/s13054-015-0751-x (PMC4344774; doi:10.1186/s13054-015-0751-x)
Supplement: Additional file 3: — Serum lactate in sham-operated and CLP-animals after 24 and 12 hours. [file 13054_2015_751_MOESM3_ESM.pdf]

Additional file 3

Serum lactate in sham-operated and CLP-animals after 24 and 12 h

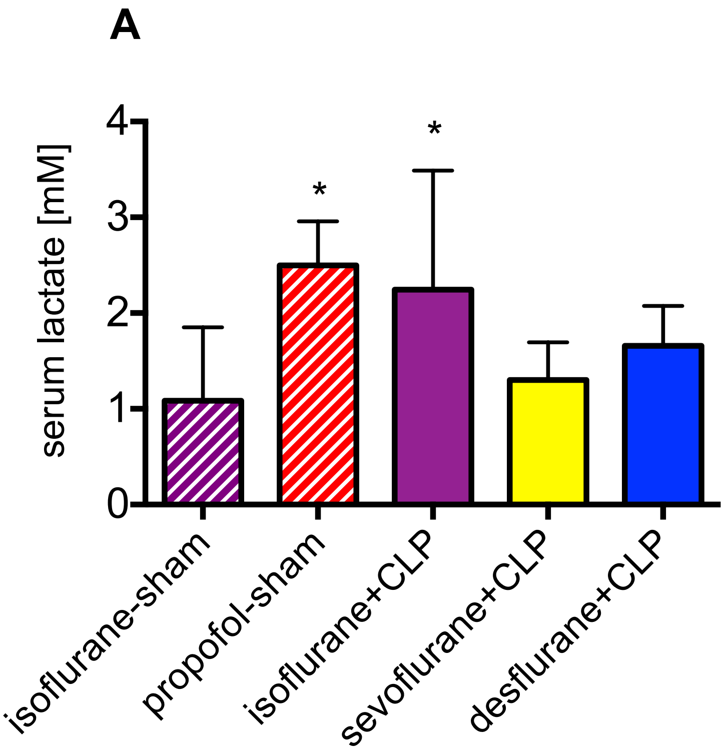

B

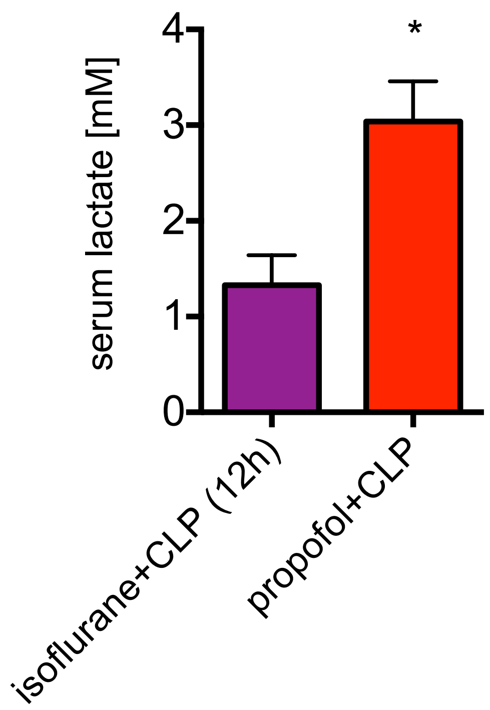

Effect of continuous sedation with propofol, isoflurane, sevoflurane or desflurane on serum lactate levels in septic (CLP) and sham-operated rats. Animals were under continuous sedation and mechanical ventilation for 24 h (A) or 12 h (B). Values represent ± standard deviation. *p<0.05 vs. isoflurane-sham (A) or isoflurane+CLP (B).
