# Supplementary material for: Propofol increases morbidity and mortality in a rat model of sepsis
Source: Crit Care. 2015 Feb 19;19(1):45. doi: 10.1186/s13054-015-0751-x (PMC4344774; doi:10.1186/s13054-015-0751-x)
Supplement: Additional file 4: — Blood oxygenation index (Horowitz index, (mmHg), mean ± standard deviation). [file 13054_2015_751_MOESM4_ESM.pdf]

Additional file 4

Blood oxygenation index (Horowitz index, [mmHg], mean ± standard deviation)

	0h	6h	12h	18h	24h
isoflurane-sham	398±83	409±111	430±79	384±111	348±112
propofol-sham	448±78	331±65	358±104	375±67	353±60
isoflurane+CLP	433±73	508±68	470±80	486±71	467±96
sevoflurane+CLP	432±78	432±117	445±95	405±94	412±140
desflurane+CLP	448±69	462±87	384±101	394±104	347±166
propofol+CLP	437±135	422±104	340±164*	403±95	388

*p<0.05 vs. sham-isoflurane

Effect of continuous sedation with propofol, isoflurane, sevoflurane or desflurane on blood oxygenation index in septic (CLP) and sham-operated rats. Blood oxygenation index was calculated by dividing PaO_2 by FiO_2.
