# Supplementary material for: Propofol increases morbidity and mortality in a rat model of sepsis
Source: Crit Care. 2015 Feb 19;19(1):45. doi: 10.1186/s13054-015-0751-x (PMC4344774; doi:10.1186/s13054-015-0751-x)
Supplement: Additional file 5: — Individual time trajectories of heart-rate in sham-operated and CLP-animals. [file 13054_2015_751_MOESM5_ESM.pdf]

Additional file 5

Individual time trajectories of heart-rate in sham-operated and CLP-animals

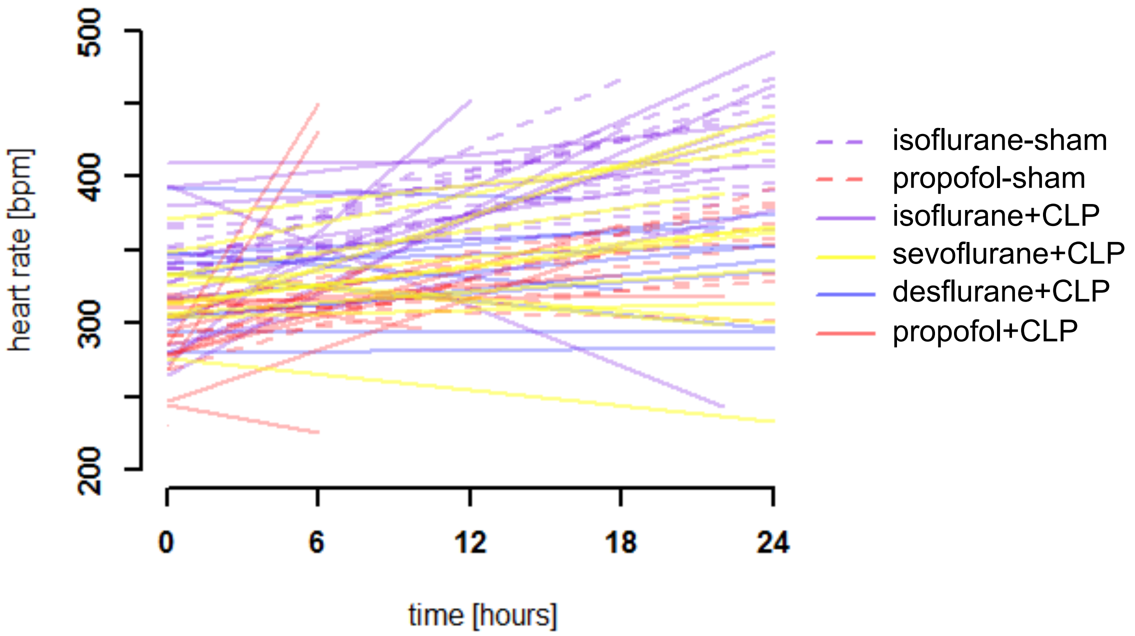

Linear regression of heart rate (in beats per minute, bpm) of septic (CLP) and sham-operated animals under continuous sedation with propofol, isoflurane, sevoflurane and desflurane displayed as individual time trajectories.
