# Supplementary material for: Propofol increases morbidity and mortality in a rat model of sepsis
Source: Crit Care. 2015 Feb 19;19(1):45. doi: 10.1186/s13054-015-0751-x (PMC4344774; doi:10.1186/s13054-015-0751-x)
Supplement: Additional file 6: — Individual time trajectories of mean arterial pressure in sham-operated and CLP-animals. [file 13054_2015_751_MOESM6_ESM.pdf]

Additional file 6

Individual time trajectories of mean arterial pressure in sham-operated and CLP-animals

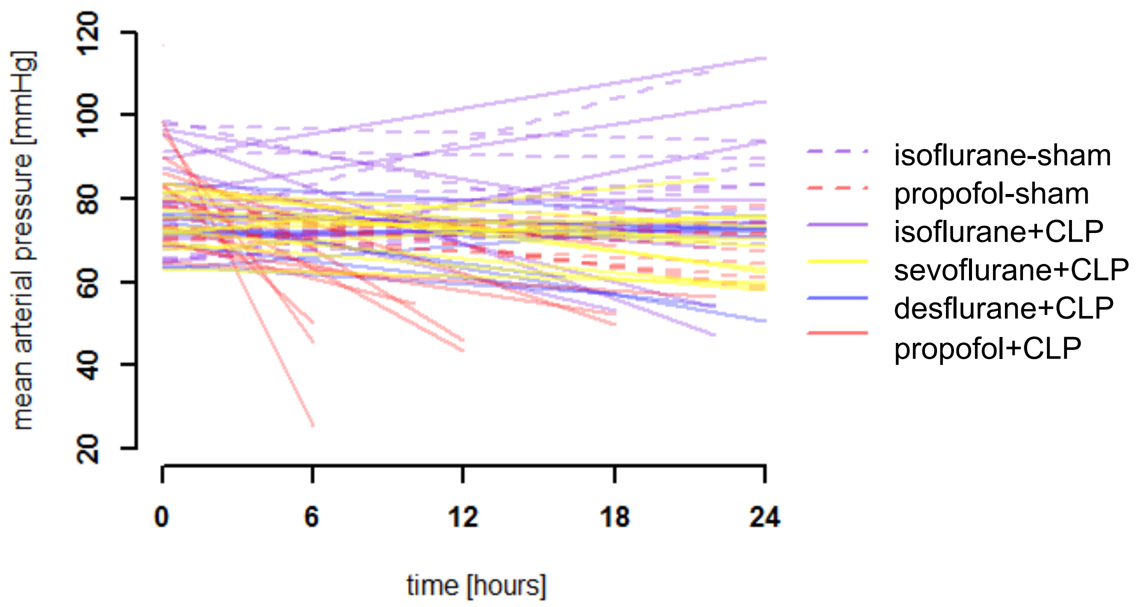

Linear regression of mean arterial blood pressure of septic (CLP) and sham-operated animals under continuous sedation with propofol, isoflurane, sevoflurane and desflurane displayed as individual time trajectories.
