# Supplementary material for: Propofol increases morbidity and mortality in a rat model of sepsis
Source: Crit Care. 2015 Feb 19;19(1):45. doi: 10.1186/s13054-015-0751-x (PMC4344774; doi:10.1186/s13054-015-0751-x)
Supplement: Additional file 7: — Influence of intralipid on serum endotoxin levels. [file 13054_2015_751_MOESM7_ESM.pdf]

Additional file 7

Influence of intralipid on serum endotoxin levels

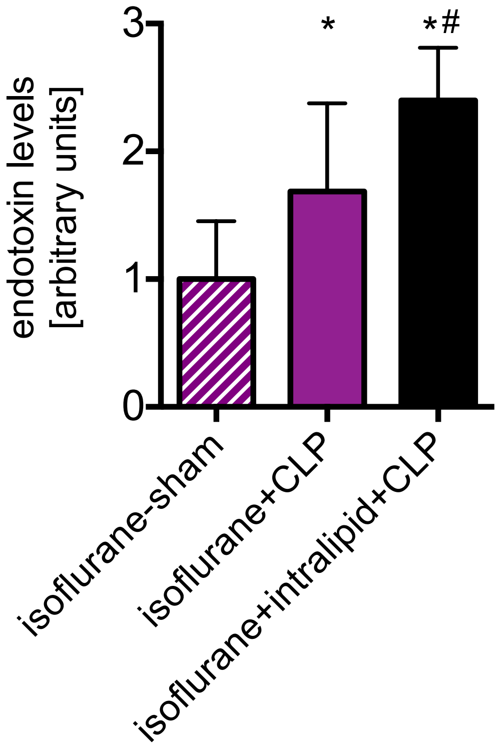

Effect of intralipid on blood endotoxin levels of septic (CLP) animals under continuous isoflurane sedation. Values are presented as mean ± standard deviation. *$p<0.05$ vs. isoflurane-sham, #$p<0.05$ vs. isoflurane+CLP.
