# Supplementary material for: Propofol increases morbidity and mortality in a rat model of sepsis
Source: Crit Care. 2015 Feb 19;19(1):45. doi: 10.1186/s13054-015-0751-x (PMC4344774; doi:10.1186/s13054-015-0751-x)
Supplement: Additional file 8: — Influence of sedatives on creatine kinase in sepsis. [file 13054_2015_751_MOESM8_ESM.pdf]

Additional file 8

Influence of sedatives on creatine kinase in sepsis

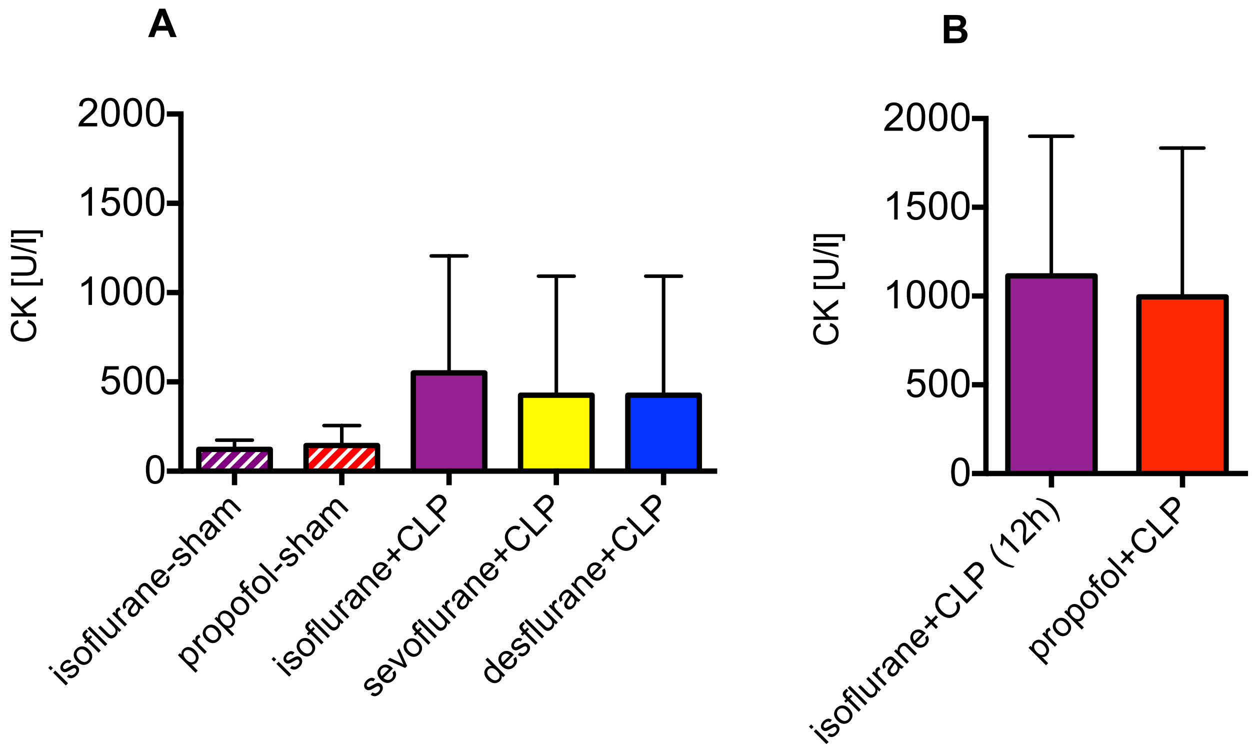

Effect of sedatives and sepsis on creatine kinase (CK) levels in the blood after 24h (A) and 12h (B) respectively. Values are presented as mean ± standard deviation in units per liter (U/l).
