# Supplementary material for: Propofol increases morbidity and mortality in a rat model of sepsis
Source: Crit Care. 2015 Feb 19;19(1):45. doi: 10.1186/s13054-015-0751-x (PMC4344774; doi:10.1186/s13054-015-0751-x)
Supplement: Additional file 9: — Serum NOx levels in sham-operated and CLP-animals. [file 13054_2015_751_MOESM9_ESM.pdf]

Additional file 9

Serum NOx levels in sham-operated and CLP-animals

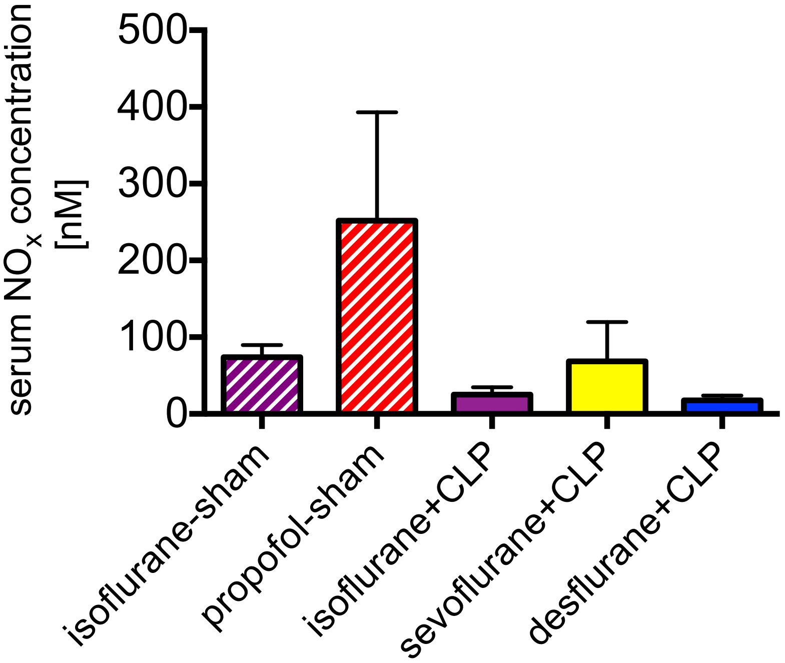

Effect of continuous sedation with propofol, isoflurane, sevoflurane or desflurane on serum nitrogen oxide (NOx) levels in septic (CLP) and sham-operated rats. Values represent ± standard deviation.
