# Supplementary material for: Propofol increases morbidity and mortality in a rat model of sepsis
Source: Crit Care. 2015 Feb 19;19(1):45. doi: 10.1186/s13054-015-0751-x (PMC4344774; doi:10.1186/s13054-015-0751-x)
Supplement: Additional file 10: — Amount of mitochondrial complex IV in septic animals after 12 hours. [file 13054_2015_751_MOESM10_ESM.pdf]

Additional file 10

Amount of mitochondrial complex IV in septic animals after 12 h

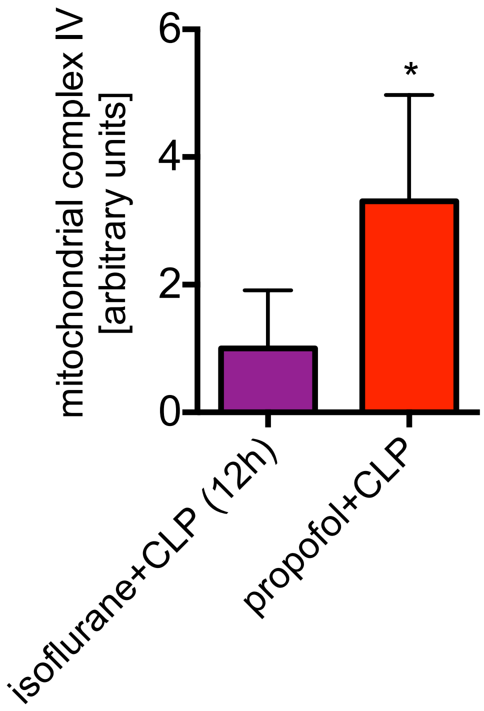

Effect of continuous sedation with propofol or isoflurane on cardiac mitochondrial complex IV in septic (CLP) rats after 12 h. Vaules represent ± standard deviation. *p<0.05 vs. isoflurane+CLP (12 h).
